# Implication of 4E-BP1 protein dephosphorylation and accumulation in pancreatic cancer cell death induced by combined gemcitabine and TRAIL

**DOI:** 10.1038/s41419-017-0001-z

**Published:** 2017-12-12

**Authors:** Androulla Elia, Ricky Henry-Grant, Charlotte Adiseshiah, Catherine Marboeuf, Rebecca J Buckley, Michael J Clemens, Satvinder Mudan, Stéphane Pyronnet

**Affiliations:** 1 Translational Control Group, Molecular and Clinical Sciences Research Institute, St George’s, University of London, Cranmer Terrace, London, SW17 0RE UK; 2 INSERM UMR-1037, Cancer Research Center of Toulouse (CRCT), Equipe Labellisée Ligue Contre le Cancer and Laboratoire d’Excellence Toulouse Cancer (TOUCAN), 31037 Toulouse, France; 3 Reproductive and Cardiovascular Disease Research Group, Molecular and Clinical Sciences Research Institute, St. George’s, University of London, Cranmer Terrace, London, SW17 0RE UK; 4 Department of Biochemistry and Molecular Biology, School of Life Sciences, University of Sussex, Falmer, Brighton, BN1 9QG UK; 5 Department of Surgery, Royal Marsden Hospital, Fulham Road, London, SW3 6JJ UK

## Abstract

Pancreatic cancer cells show varying sensitivity to the anticancer effects of gemcitabine. However, as a chemotherapeutic agent, gemcitabine can cause intolerably high levels of toxicity and patients often develop resistance to the beneficial effects of this drug. Combination studies show that use of gemcitabine with the pro-apoptotic cytokine TRAIL can enhance the inhibition of survival and induction of apoptosis of pancreatic cancer cells. Additionally, following combination treatment there is a dramatic increase in the level of the hypophosphorylated form of the tumour suppressor protein 4E-BP1. This is associated with inhibition of mTOR activity, resulting from caspase-mediated cleavage of the Raptor and Rictor components of mTOR. Use of the pan-caspase inhibitor Z-VAD-FMK indicates that the increase in level of 4E-BP1 is also caspase-mediated. ShRNA-silencing of 4E-BP1 expression renders cells more resistant to cell death induced by the combination treatment. Since the levels of 4E-BP1 are relatively low in untreated pancreatic cancer cells these results suggest that combined therapy with gemcitabine and TRAIL could improve the responsiveness of tumours to treatment by elevating the expression of 4E-BP1.

## Introduction

Pancreatic ductal adenocarcinoma (PDAC) is an aggressive cancer with 5-year survival rates that have remained at only about 5%^1,2^. The disease is often only detected at a late stage but, additionally, tumours are commonly resistant to conventional therapies^3^. As a single agent, the nucleoside analogue gemcitabine has been the standard treatment for pancreatic cancer for several years, and patients have been shown to have an improved quality of life following therapy^4^. However, the development of resistance to treatment presents an urgent need for novel strategies, including the identification of agents that can enhance the effect of gemcitabine at doses that have low toxicity^5,6^. In many cancers the protein kinase mammalian target of rapamycin (mTOR) is hyperactivated, leading to an increase in the phosphorylation of several downstream targets^7,8^. One such target is the tumour suppressor 4E-BP1. In its hypophosphorylated form 4E-BP1 functions as a binding protein that regulates the availability of the oncogenic polypeptide chain initiation factor eIF4E during the initiation of protein synthesis^9,10^. Previous studies have shown that in some pancreatic cancer cells 4E-BP1 is expressed at very low levels and that the protein is highly phosphorylated^11^. Indeed, the levels of phosphorylated 4E-BP1 have been used as a prognostic indicator in a number of cancer types^12–16^.

Many studies have established that the levels of eIF4E are elevated in a number of malignancies and that excessive expression of eIF4E is oncogenic due to its ability to confer resistance to apoptosis^17–24^. Conversely, the dephosphorylated form of 4E-BP1 has pro-apoptotic effects^25,26^. There is a correlation between the extent of phosphorylation of 4E-BP1 and the state of aggressiveness of tumours^27,28^, and changes in the levels of the tumour suppressor can affect the ability of malignant cells to undergo apoptosis^29,30^.

A better understanding of cancer immunotherapy has identified the tumour necrosis factor-related apoptosis-inducing ligand (TRAIL) as a cytokine with the ability to target cancer cells whilst sparing non-malignant cells. This property indicates that TRAIL has the potential to be an important anticancer agent^31,32^. TRAIL induces extrinsic apoptosis by binding to either of two death receptors (DRs), TRAIL-R1/DR4 and TRAIL-R2/DR5. However, recent work indicates that many cancer cell lines are resistant to TRAIL treatment and this has limited its therapeutic use^33^. In fact, several clinical trials using soluble forms of TRAIL such as dulanerim have proved disappointing^34,35^. With the emergence of newer and more stable forms of TRAIL, coupled with more efficient delivery methods, the potential for more effective therapies looks promising^36,37^. Relatively few studies have thus far focused on the possible use of combination therapy using gemcitabine together with TRAIL^38–40^.

We have previously investigated the role of 4E-BP1 in regulating the sensitivity of pancreatic cancer cells to TRAIL-induced apoptosis^29^. However, the possible importance of 4E-BP1 in determining the effectiveness of TRAIL in combination with gemcitabine has not been addressed. In this study we have used soluble recombinant human TRAIL in combination with gemcitabine to investigate possible effects on the regulation of apoptosis in pancreatic cancer cells. We demonstrate that the use of gemcitabine and TRAIL enhances the inhibition of survival of pancreatic cancer cells and provide data to show that both the extent of dephosphorylation and the level of total 4E-BP1 are strongly increased as a result of the combination treatment. These changes are associated with an inhibition of mTOR activity and caspase-mediated cleavage of the Raptor and Rictor components of mTOR. Reducing the expression of 4E-BP1 using small hairpin RNAs (shRNAs) impairs the induction of cell death following combination treatment of the pancreatic cancer cells. Possible mechanisms by which 4E-BP1 functions as an important determinant of the sensitivity of pancreatic cancer cells to cell death effects of gemcitabine and TRAIL are discussed.

## Results

### Cytotoxic effects of gemcitabine and TRAIL treatment on human pancreatic cancer cells

**Fig. 1:**
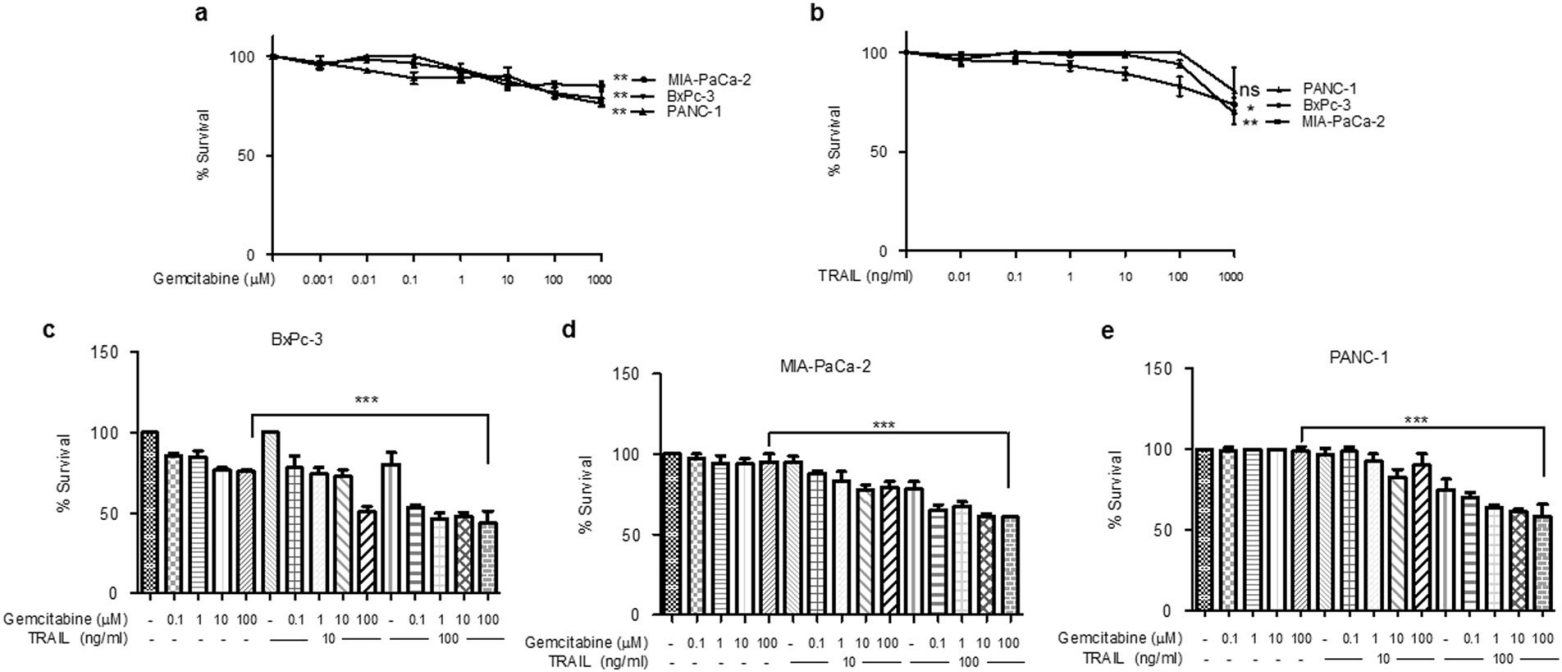
Effect of gemcitabine and/or TRAIL on PDAC survival. BxPC-3, MIA PaCa-2 and PANC-1 cells were seeded in 96-well plates at a cell seeding density of 3x10^4^ cells/cm^2^ **a** Sensitivity of cells to gemcitabine was assessed by MTT assay. Cells were treated with increasing amounts of gemcitabine (0.001–1000 μM) for 24 h (*n *= 4). **b** Sensitivity of cells to TRAIL was assessed by MTT assay. Cells were treated with increasing amounts of TRAIL (0.001–1000 ng/ml) for 4 h (*n* = 4). **c**–**e** Sensitivity of cells to gemcitabine and TRAIL combination treatment was assessed by MTT assay. Cells were treated with increasing amounts of gemcitabine (0.1–100 μM) for 24 h (*n* = 4) and/or 10 or 100 ng/ml TRAIL for 4 h for BxPC-3 and MIA PaCa-2 cells and 6 h for PANC-1 cells (*n* = 4). All experiments were repeated three times and data are provided as means ± SEM (one representative experiment is shown). *P*-values were calculated using Student’s *t*-test to determine the statistical significance of the difference between **a**, **b** untreated cells and cells treated with either 1000 μM gemcitabine or 1000 ng/ml TRAIL, respectively (ns: *P* > 0.05, *P < 0.05, **P < 0.01) and **c**–**e** cells treated with 100 μM gemcitabine and cells treated with 100 μM gemcitabine plus 100 ng/ml TRAIL (***P < 0.001)

As gemcitabine is widely used as a first-line chemotherapeutic drug in the treatment of pancreatic cancer, characterisation of its cytotoxic effects has been widely reported^41–43^. Using the thiazolyl blue tetrazolium bromide (MTT) assay we have extended these studies to examine the effects of gemcitabine in combination with TRAIL in three PDAC cell lines: BxPC-3; MIA PaCa-2; and PANC-1. All three cell lines exhibited relatively poor sensitivity to the cytotoxic effects of gemcitabine alone after 24 h exposure to concentrations up to 1000 μM (Fig. 1a). In parallel with these assays we tested the sensitivities of the cell lines to TRAIL alone. MIA PaCa-2 cells were the most sensitive to treatment and exposure to a concentration of 10 ng/ml TRAIL significantly inhibited their survival. BxPC-3 were resistant to TRAIL at up to 100 ng/ml (4 h treatment), and there was no significant effect of TRAIL on the survival of PANC-1 cells even at a 10-fold higher concentration (Fig. 1b).

We then examined whether co-treatment of the cells with both reagents could result in a more significant inhibition of survival. The MTT assays showed that a treatment using 100 μM gemcitabine in combination with 100 ng/ml TRAIL significantly inhibited cell survival in all three cell types (Fig. 1c–e). For example, whereas 100 μM gemcitabine alone had only effects of 24.3% and 4.9% on BxPC-3 and MIA PaCa-2 cells, respectively, in the presence of TRAIL at 100 ng/ml for 4 h the inhibitory effects of gemcitabine were increased to 56.5% and 39.2%. As the PANC-1 cell line was less responsive to TRAIL, we extended the treatment time to 6 h and were able to show similar effects in these cells too (Fig. 1e and Supplementary Fig. S1a).

### Gemcitabine enhances TRAIL-induced apoptosis

**Fig. 2:**
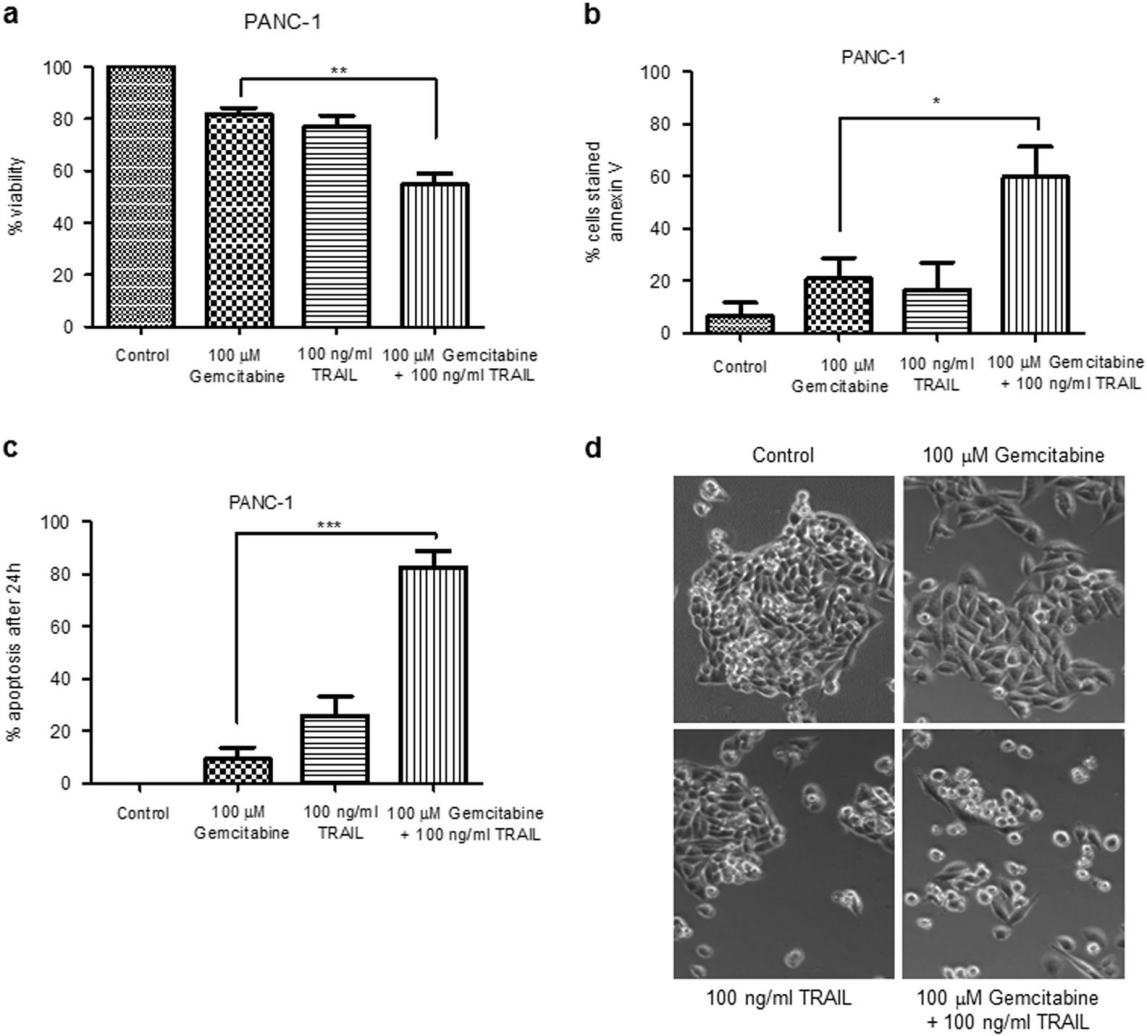
Combination treatment induces apoptosis **a** PANC-1 cells were seeded in triplicate in 12-well plates at a cell seeding density of 3×10^4^ cells/cm^2^ and left to attach overnight. Cells were treated with 100 μM gemcitabine for 24 h and/or 100 ng/ml TRAIL for 6 h. The viability of the cells was assessed by trypan blue exclusion assay. Quadruplicate cell counts were used to calculate each cell density. These were performed for three independently seeded wells and percentage viability was determined. **b** A total of 1×10^6^ PANC-1 cells were treated with 100 μM gemcitabine for 24 h and/or 100 ng/ml TRAIL for 6 h. Induction of early apoptosis in PANC-1 cells was assessed using flow cytometry following staining with FITC Annexin V. The data represent means ± SEM of three experiments performed in triplicate. **c**, **d** PANC-1 cells were seeded in triplicate in 12-well plates at a cell seeding density of 3×10^4^ cells/cm^2^ and left to attach overnight. Cells were treated with 100 μM gemcitabine for 24 h and/or 100 ng/ml TRAIL for 6 h and monitored by time-lapse microscopy. **c** The appearance of a pre-apoptotic morphology was scored and the % apoptotic cells after 24 h determined. The data are the means ± SEM from three independent experiments. **d** Phase contrast microscopy images of cells treated as indicated. **a**–**c** All experiments were repeated three times and data are provided as means ± SEM (one representative experiment is shown). *P*-values were calculated using Student’s *t*-test to determine the statistical significance of the difference between cells treated with 100 μM gemcitabine and cells treated with 100 μM gemcitabine plus 100 ng/ml TRAIL (*P < 0.05, **P < 0.01, ***P < 0.001)

Since TRAIL is a well-known inducer of apoptosis we used the trypan blue exclusion assay to assess the effect of co-treatment with gemcitabine on cell viability. Even in the case of PANC-1 cells, the least responsive of the cell types, 100 μM gemcitabine in combination with 100 ng/ml TRAIL significantly inhibited viability, reducing it by 43.8%, whereas either agent alone was much less effective (Fig. 2a).

The induction of apoptosis following combination treatment of the PANC-1 cells was monitored using a variety of methods. Using flow cytometry we observed that combination treatment of PANC-1 cells resulted in significantly enhanced externalisation of phosphatidylserine (measured by Annexin V binding) compared to the treatments with gemcitabine or TRAIL alone (Fig. 2b). Time-lapse microscopy was used to assess morphological changes over time and to measure the % of cells that become committed to apoptosis (Fig. 2c, d). Figure 2c demonstrates that after a 24 h period of treatment with 100 μM gemcitabine in combination with 100 ng/ml TRAIL, 82.5% of PANC-1 cells had undergone complete apoptosis, significantly much higher than with the individual treatments alone.

**Fig. 3:**
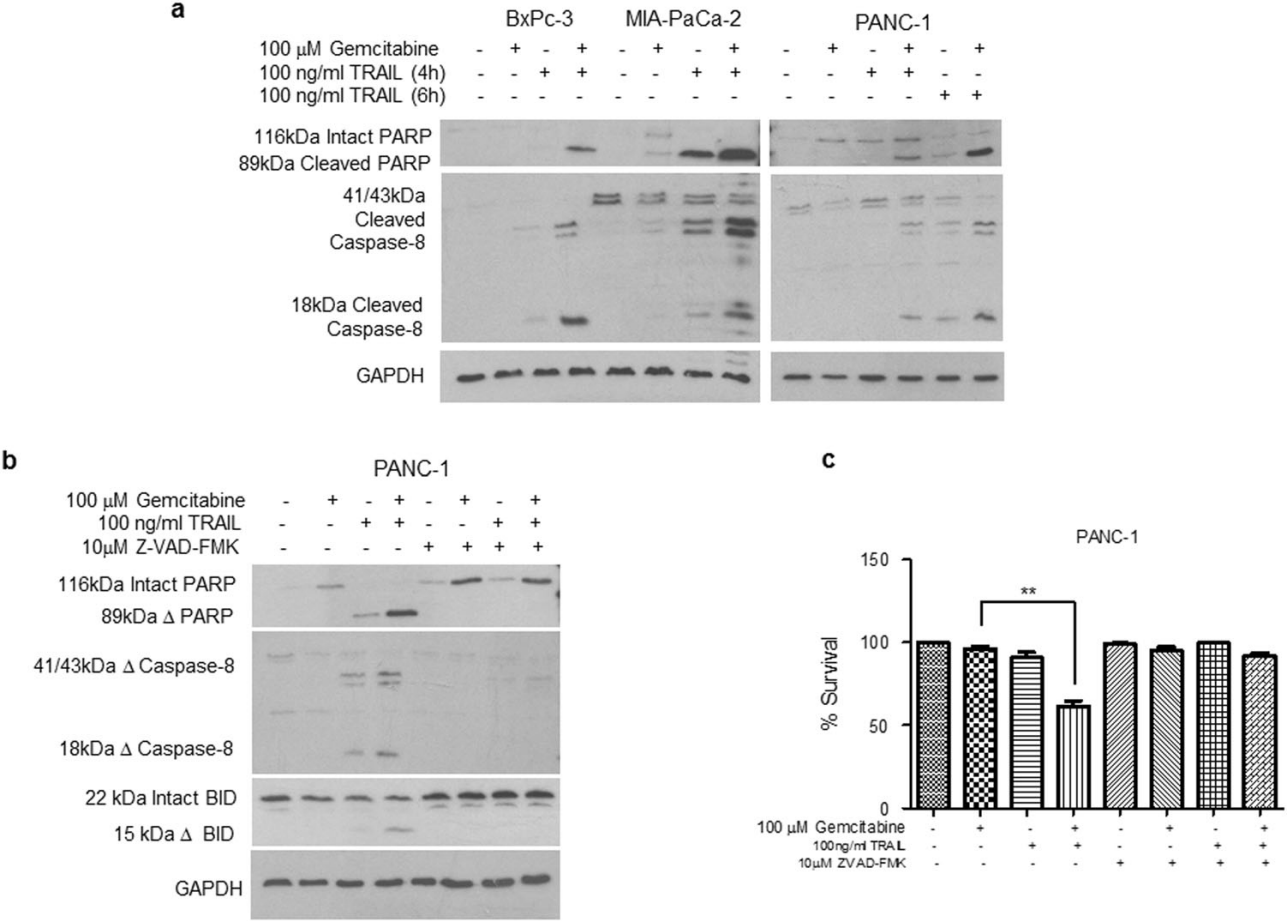
Combination treatment induces caspase-dependent apoptosis. BxPC-3, MIA PaCa-2 and PANC-1 cells were seeded in 96-well plates at a cell seeding density of 3×10^4^ cells/cm^2^ **a**, **b** Caspase-mediated cleavage of caspase-8 and PARP was assessed by western blotting in cells treated with 100 μΜ gemcitabine for 24 h and/or 100 ng/ml TRAIL for 4 h for BxPC-3 and MIA PaCa-2 cells, and 4 and 6 h for PANC-1 cells (*n *= 3). One representative experiment is shown. Lysates were prepared and equal amounts (15 μg total protein) were subjected to SDS–PAGE, transferred to PVDF membranes and then immunoblotted with antibodies directed against **a** PARP (top panel), caspase-8 (middle panel) or GAPDH (bottom panel). **b** Caspase-mediated cleavages of caspase-8, PARP and BID in the presence or absence of the pan-caspase inhibitor Z-VAD-FMK (10 μM) were assessed by western blotting in cells treated as described above. Membranes were immunoblotted with antibodies directed against caspase-8, PARP and BID. GAPDH was used as a loading control. **c** The inhibition of cell survival following combination treatment was assessed in the presence or absence of the pan-caspase inhibitor Z-VAD-FMK. PANC-1 cells were seeded in 96-well plates at a cell seeding density of 3×10^4^ cells/cm^2^. Cells were treated with 100 μM gemcitabine for 24 h and/or 100 ng/ml TRAIL for 6 h in the presence or absence of 10 μM Z-VAD-FMK. Cell survival was assessed using the MTT assay. All experiments were repeated three times and data are provided as means ± SEM (one representative experiment is shown). *P*-values were calculated using Student’s *t*-test to determine the statistical significance of the difference between cells treated with 100 μM gemcitabine and those treated with both 100 μM gemcitabine and 100 ng/ml TRAIL (*P < 0.05, **P < 0.01, ***P < 0.001)

We further examined the ability of the combination therapy to enhance apoptosis using western blotting to determine the cleavage of caspase-8 and poly(ADP-ribose) polymerase (PARP) (Fig. 3a). All three cell lines showed enhanced cleavage of both caspase substrates following the combination treatment, with PANC-1 cells exhibiting virtually complete cleavages at 6 h. Additionally, we observed cleavage of BID, a BH3 domain-containing pro-apoptotic Bcl2 family member in PANC-1 cells (Fig. 3b). Such cleavage releases a potent pro-apoptotic activity of BID and provides a critical link between the activation of caspase-8 and the intrinsic apoptotic pathway^44^.

Both the inhibition of survival and the induction of apoptosis following combination treatment were caspase-dependent as these effects were blocked by the addition of the pan-caspase inhibitor Z-VAD-FMK (Fig. 3b, c).

### Effects of gemcitabine and TRAIL on the mTOR pathway

**Fig. 4:**
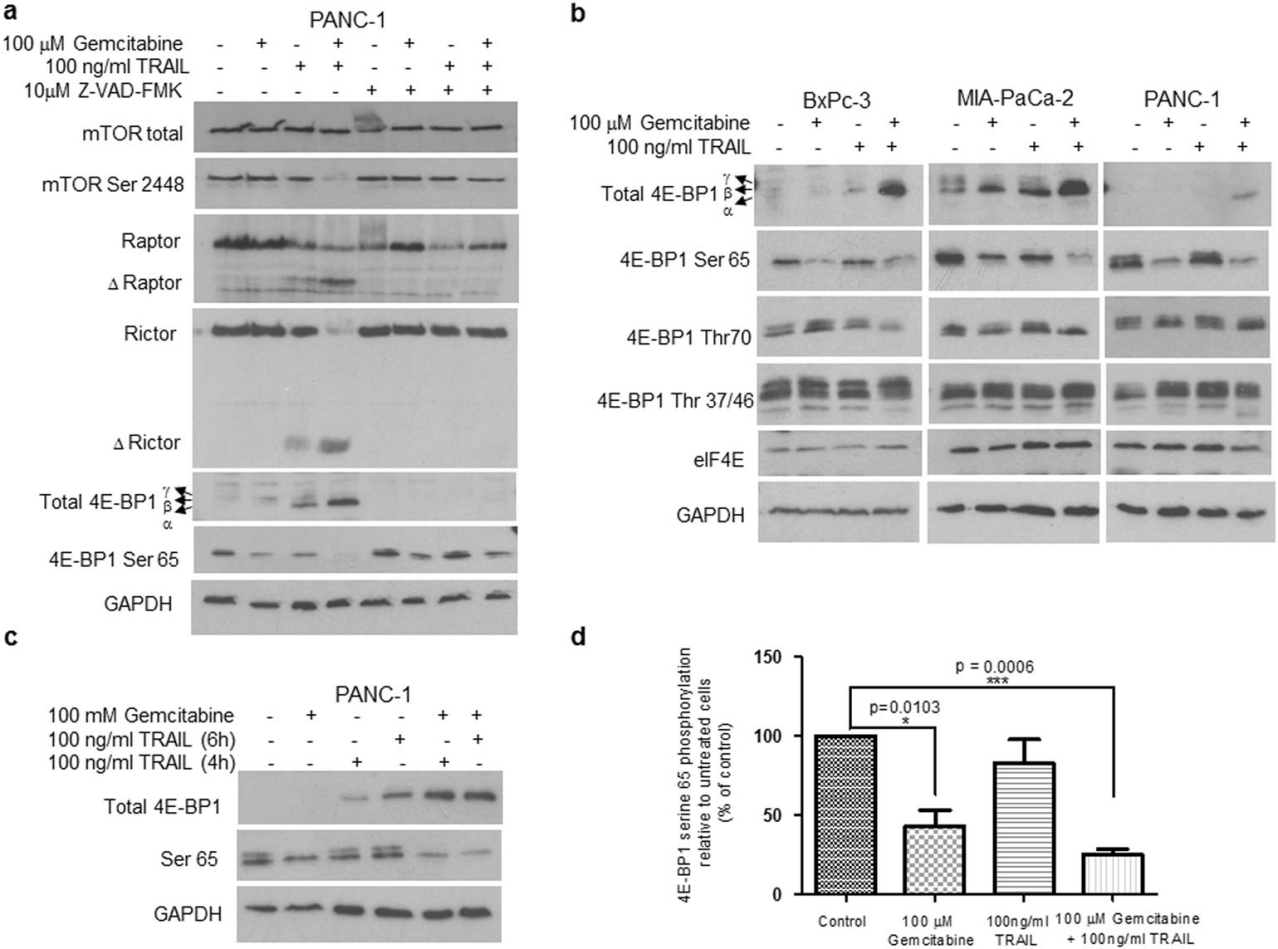
Combination treatment targets the mTOR pathway and alters the phosphorylation of 4E-BP1 in PDAC cells BxPC-3, MIA PaCa-2 cells and PANC-1 cells were treated with 100 μΜ gemcitabine for 24 h and/or 100 ng/ml TRAIL for 4 h. A unit of 15 μg of total protein lysate was analysed using western blotting. **a** PANC-1 cell lysates were analysed with antibodies directed against total mTOR, mTOR Ser^2448^, Raptor, Rictor, total 4E-BP1, 4E-BP1 Ser^65^ and GAPDH. **b** BxPC-3, MIA PaCA-2 and PANC-1 lysates were analysed to look at the effect on levels and phosphorylation of 4E-BP1 at residues Ser^65^, Thr ^37/46^ and Thr^70^ as well as levels of eIF4E. GAPDH was used as a loading control. **c** The change in phosphorylation of 4E-BP1 at Ser^65^ in PANC-1 cells following combination treatment using TRAIL treatment for either 4 or 6 h was assessed by western blotting. PVDF membranes were immunoblotted with antibodies directed against total 4E-BP1 and 4E-BP1 residue Ser^65^. **d** The relative levels of phosphorylation of 4E-BP1 at Ser^65^ were quantified by scanning densitometry using ImageJ and the data are shown on the histogram as % of the values for untreated cells. All experiments were repeated three times and data are provided as means ± SEM. *P*-values were calculated using Student’s *t*-test to determine the statistical significance of the difference between untreated cells and cells treated with either gemcitabine or gemcitabine plus TRAIL (*P < 0.05 and ***P < 0.001)

The pharmacological targeting of the mTOR/4E-BP1 pathway in pancreatic cancer has been previously reported^45^. In order to investigate whether the pathway is involved in the inhibition of survival and pro-apoptotic effects of gemcitabine and TRAIL on PDAC cells, we characterised the effects of these agents on various aspects of the mTOR pathway. The effects of the gemcitabine and TRAIL combination were apparent at the level of phosphorylation of mTOR itself, which showed dephosphorylation at Ser^2448^ in PANC-1 cell (Fig. 4a). In addition, there was TRAIL-mediated and caspase-dependent cleavage of the proteins Raptor and Rictor, which are associated with the mTORC-1 and mTORC-2 complexes, respectively (Fig. 4a). Similar effects on the level of phosphorylation of mTOR- and caspase-mediated cleavage of Raptor and Rictor were also observed in BxPC-3 and MIA PaCa-2 cells (Supplementary Fig. 2a, b).

We have previously shown that TRAIL can cause the dephosphorylation of the mTOR substrate 4E-BP1 in pancreatic cancer cells^29^. However, the effect of the cytokine when used in combination with gemcitabine on the phosphorylation state of 4E-BP1 has not previously been investigated. Extracts made from the three cell lines were analysed by western blotting using either antibodies to total 4E-BP1 or three phospho-specific antibodies recognising the phosphorylation sites Ser^65^, Thr^37/46^ or Thr^70^ (Fig. 4b, c).

With the exception of MIA PaCa-2 cells there was very little effect on the levels of total 4E-BP1 following the individual treatments (in PANC-1 cells very little 4E-BP1 could be detected under these conditions) (Fig. 4b). TRAIL treatment alone had no significant effect on phosphorylation of 4E-BP1 at any of the sites investigated. Interestingly, gemcitabine alone caused dephosphorylation of 4E-BP1 at Ser^65^ in all three cell lines (Fig. 4b, c). In PANC-1 cells this dephosphorylation was observed despite negligible levels of total protein being detectable (Fig. 4b, c). Gemcitabine treatment of PANC-1 cells resulted in a significant 57% reduction in phosphorylation of 4E-BP1 at Ser^65^, whereas gemcitabine plus TRAIL resulted in a 74.6% reduction (Fig. 4d).

The most dramatic changes in the levels and phosphorylation of 4E-BP1 followed combination treatment of the cells, where a marked elevation in the levels of total 4E-BP1 was observed in all three cell lines (particularly BxPC-3 and MIA PaCa-2) (Fig. 4b). Additionally, all cell types exhibited strong dephosphorylation of Ser^65^ in response to gemcitabine plus TRAIL (Fig. 4b, c). Dephosphorylation at the other sites was observed but is only apparent when the large increases in total levels of 4E-BP1 are taken into account. The substantial increase in the level of total 4E-BP1 is of considerable interest in view of the fact that 4E-BP1 expression is severely repressed in a high proportion of human pancreatic tumours^11^. As we did not observe any changes in the levels of the potentially oncogenic factor eIF4E following treatment (Fig. 4b), the ratio of 4E-BP1 to eIF4E becomes much higher after gemcitabine and TRAIL treatment and it is therefore not surprising that there was a marked inhibition of protein synthesis (Supplementary Fig. S1b, c, d and data not shown). Using protein synthesis assays we determined that MIA PaCa-2 cells treated with 100 μM gemcitabine in combination with 100 ng/ml TRAIL for 4 h showed a 73% inhibition of protein synthesis compared to 45% inhibition following treatment of the cells with 100 μM gemcitabine in combination with 10 ng/ml TRAIL for 4h.

Consistent with the above findings, the use of m^7^GTP-Sepharose affinity chromatography to purify eIF4E and its associated proteins demonstrated a large increase in the binding of 4E-BP1 to eIF4E in PANC-1 cells treated with 100 μM gemcitabine in combination with 100 ng/ml TRAIL for 6 h (Supplementary Fig. S1b).

Since TRAIL enhances caspase activity in its target cells we investigated the caspase-dependence of the effects of this combination treatment, using the board specific caspase inhibitor Z-VAD-FMK. Interestingly, both the increases in levels of 4E-BP1 and the dephosphorylation of 4E-BP1 and mTOR described above require caspase activity as pre-treatment of the cells with the pan-caspase inhibitor Z-VAD-FMK was able to prevent these effects (Fig. 4a).

### Role of 4E-BP1 in the cytotoxic effects of gemcitabine and TRAIL

Following on from the above data, we investigated whether 4E-BP1 plays a required role in the regulation of survival of PDAC cells by the combination of gemcitabine and TRAIL. For this purpose as the MIA PaCa-2 cell line is the only cell line, which expresses constitutive high levels of 4E-BP1 while eIF4E is equally expressed in the three (Fig. 4b)^46^, we employed two stable MIA PaCa-2 cell lines engineered to express either shRNA directed against 4E-BP1 or scrambled shRNA as a control^47^.

**Fig. 5:**
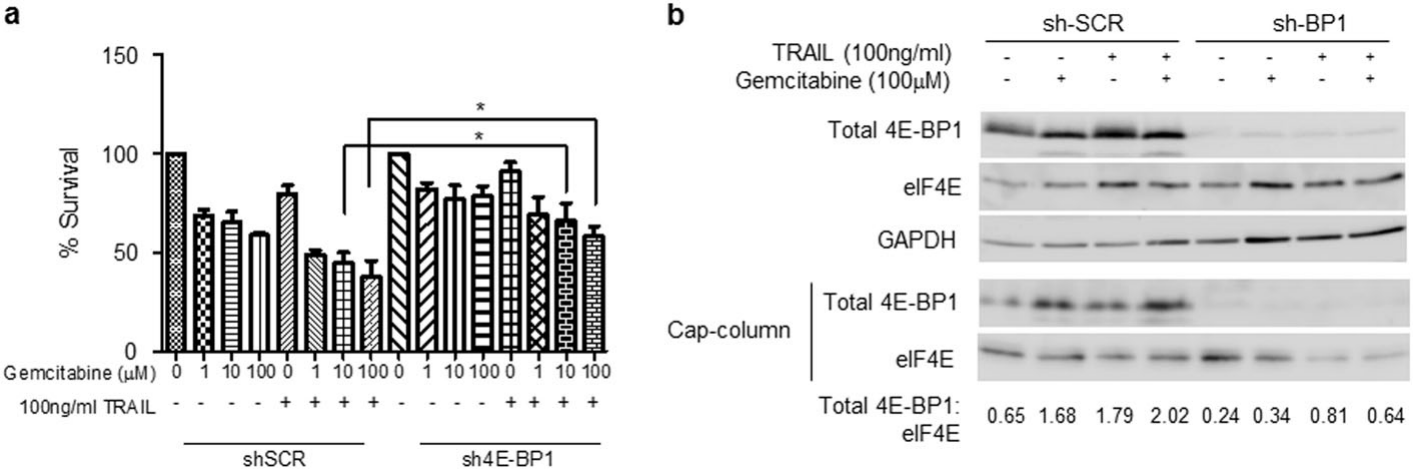
4E-BP1 is involved in the regulation of cell survival following gemcitabine and TRAIL treatment **a**, **b** MIA PaCa-2 cells expressing a small hairpin RNA (shRNA) directed against 4E-BP1 and control cells expressing a scrambled shRNA were seeded in 96-well plates at a cell seeding density of 3×10^4^ cells/cm^2^. **a** The sensitivity of cells to gemcitabine and TRAIL combination treatment was assessed by MTT assay. Cells were treated with increasing amounts of gemcitabine (0.1–100 μM) for 24 h (*n* = 4) and/or 100 ng/ml TRAIL for 24 h (*n *= 4). All experiments were repeated three times and data are provided as means ± SEM. One representative experiment is shown. *P*-values were calculated using Student’s *t*-test to determine the statistical significance of the difference between cells expressing a scrambled shRNA and cells expressing a shRNA directed against 4E-BP1, both cell lines having been treated with 10 or 100 μM gemcitabine and 100 ng/ml TRAIL (*P < 0.05). **b** Lysates made from cells treated as in **a** were used to purify eIF4E using chromatography on m^7^GTP-Sepharose beads as described in Methods. The levels of eIF4E and of the 4E-BP1 associated with it were determined by SDS gel electrophoresis and immunoblotting. Total cell lysates were analysed in parallel. Quantification was carried out by densitometry using ImageJ and the ratios of 4E-BP1 to eIF4E in the m^7^GTP-purified samples (in arbitrary units) are indicated

In contrast to the MIA PaCa-2 cells used in our earlier work, both genetically modified cell types were resistant to TRAIL alone (Supplementary Fig. S3a), likely due to acquired changes during the process of stable cell line selection. Furthermore, when we tested the combination treatment using a TRAIL treatment time of 6 h it was apparent that there was no difference between the extent of survival of the two cell types as determined by the MTT assay (Supplementary Fig. S3b). However, after an extended treatment time of 24 h with gemcitabine plus TRAIL we did observe significant resistance of the cells in which 4E-BP1 expression had been silenced (Fig. 5a), suggesting a role for the tumour suppressor protein in the longer-term effects of the combination treatment. Using m^7^GTP-Sepharose affinity chromatography we were able to demonstrate that in the cells in which 4E-BP1 had not been silenced there was an increase in the binding of dephosphorylated 4E-BP1 to eIF4E that was more apparent following combination treatment of the cells (Fig. 5b).

## Discussion

Although various trials have investigated treatments using gemcitabine in combination with a number of reagents, none of these treatments was shown to be significantly more effective than gemcitabine alone^48–51^. So despite being first approved 30 years ago, gemcitabine still remains the first-line therapy for pancreatic cancer. In this manuscript we have investigated the effect of combining gemcitabine with the cytokine TRAIL on the survival of three PDAC and two genetically modified PDAC cell lines. We have established that using TRAIL and gemcitabine in combination can significantly inhibit survival and induce apoptosis in these cells. In particular, the combination treatment was effective in the survival of the PANC-1 cell line that is highly resistant to gemcitabine treatment alone. Although all three PDAC cell lines examined showed differing sensitivities to treatment with TRAIL as previously shown^29^, it is of significance that in the presence of TRAIL the cells become responsive to concentrations of gemcitabine that alone are ineffective. Moreover, in the more gemcitabine-sensitive cell line, BxPC-3, TRAIL renders the cells responsive to much lower concentrations of gemcitabine. We used the MIA PaCa-2 cell type to establish that the combined effect of 100 μM gemcitabine together with 100 ng/ml TRAIL was synergistic in nature, at the level of total protein synthesis.

In analysing the induction of apoptosis in PDAC cells we have shown that the combination of gemcitabine and TRAIL activates a caspase-mediated mechanism that leads to the cleavage of a number of substrates, namely PARP, caspsase-8 and BID. In all PDAC cell lines tested we also identified additional new caspase targets, notably the Rictor and Raptor components of the mTORC-1 and mTORC-2 complexes of mTOR. There is recent evidence indicating that Raptor is indeed cleaved by caspases but this has never been investigated in this model^52^. TRAIL-induced cleavage of components of mTORC-1 and mTORC-2 during cell death in PDAC cells suggests treatment options targeting this pathway^53^.

Previous studies of the underlying mechanisms by which gemcitabine and TRAIL induce cell death have implicated a number of signalling molecules. We have previously shown that TRAIL can cause dephosphorylation of the regulatory protein 4E-BP1 in a number of tumour cell types^29,54,55^. However, the effects of a combination treatment using gemcitabine and TRAIL on the phosphorylation and levels of this tumour suppressor in PDAC cell lines have been overlooked until now. Our present findings suggest that gemcitabine treatment of all PDAC cell lines investigated leads to dephosphorylation of 4E-BP1 at residue Ser^65^. However, gemcitabine alone is not sufficient to induce cell death. Since there is little or no effect of gemcitabine alone on the activity of mTOR, as judged by the state of phosphorylation of residue Ser^2448^, it is likely that the effect of gemcitabine on 4E-BP1 phosphorylation is mTOR-independent. Using western blotting we were able to see a dephosphorylation of 4E-BP1 at Ser^65^ in all cell lines following treatment with 100 μM gemcitabine and 100 ng/ml TRAIL, and in the PANC-1 cells the combination treatment significantly reduced the phosphorylation of this residue compared to untreated cells. The latter effect coincides with dephosphorylation of mTOR at Ser^2448^ as well as caspase-dependent cleavages of Raptor and Rictor. Overall, these observations indicate that the combination of gemcitabine and TRAIL acts via both mTOR-dependent and -independent pathways.

In addition to the dephosphorylation of 4E-BP1 we noted very marked increases in the levels of total 4E-BP1 in all cell lines following the combination treatment. This is likely to be of considerable significance with regards to the functional activity of the protein. In PANC-1 cells binding of 4E-BP1 to eIF4E, isolated on m^7^GTP-Sepharose, was only observed at the higher levels of 4E-BP1, namely after TRAIL treatment alone or after TRAIL in combination with gemcitabine. This is likely to be of particular relevance in PDAC cells where the basal levels of 4E-BP1 are very low^11^. Taken together, these data suggest that gemcitabine leads to a dephosphorylation of 4E-BP1 but that this alone is not sufficient to induce cell death. However, gemcitabine potentiates the pro-apoptotic effect of TRAIL by a mechanism that may involve enhanced expression of 4E-BP1.

To test whether changes in the levels of 4E-BP1 play a role in determining the sensitivity of PDAC cells to the combination treatment we used a MIA PaCa-2 cell line in which 4E-BP1 can be downregulated^47^. The cell lines used for this experiment were derived from MIA PaCa-2 but proved to be much more resistant to TRAIL than the MIA PaCa-2 cells used in our other studies. Treatment of both the control and 4E-BP1-negative cells with concentrations of TRAIL up to 1000 ng/ml for 6 h had little effect on the survival of these MIA PaCa-2-derived cell lines. This may be a consequence of the selection of stable transfectants with puromycin during the development of the cell line. However, extended treatment of these cells with TRAIL for 24 h enabled us to demonstrate that in the absence of 4E-BP1 the cells were significantly more resistant to the combination treatment. The data from these experiments further suggest that the pro-apoptotic effect of TRAIL alone is not dependent on 4E-BP1 but the potentiating effect of gemcitabine is dependent on expression of the tumour suppressor.

Although in some circumstances TRAIL has been shown to promote the growth of pancreatic cancer^56^ there is extensive evidence for a physiological function of endogenous TRAIL as a tumour suppressor. The cytokine has been shown to be an important natural effector molecule in the armoury of host defences against transformed cells and it has a critical role in immune surveillance^57,58^. Whilst we have investigated the effect of combining gemcitabine with TRAIL as a basis for an improved chemotherapeutic approach, newly emerging immunotherapies targeted against pancreatic cancer that increase the levels of endogenous TRAIL may also benefit from the combined use of gemcitabine^59–61^. Endogenously expressed TRAIL is known to be several orders of magnitude more active than conventional soluble trimeric TRAIL^62^. Irrespective of either therapeutic approach, this study shows the promising potential of using a combination of gemcitabine with TRAIL as a way of re-sensitising gemcitabine-resistance PDAC cells, ultimately inducing these cells to undergo apoptosis. Our data suggest that the marked upregulation and dephosphorylation of 4E-BP1 is likely to play an important role in this promotion of cell death.

## Methods

### Materials

Tissue culture reagents were supplied by Sigma, Poole, UK. Antibody to 4E-BP1 (R113) was from Santa Cruz Biotechnology, CA, USA. Antibodies against phosphorylated 4E-BP1 (anti-Ser^65^ catalogue number 9451, anti-Thr^37/46^ catalogue number 9459 and anti-Thr^70^ catalogue number 9455), caspase-8, biotinylated gel markers and cell lysis buffer were all from Cell Signalling Technology, Hitchin, UK. Mouse anti-PARP was purchased from BD Pharmingen, Oxford, UK. The antibody to GAPDH was from Millipore, Watford, UK. All secondary antibodies (anti-rabbit-horseradish peroxidase (HRP) linked, anti-mouse-HRP linked or anti-biotin-HRP linked) were obtained from Cell Signalling Technology. Polyvinylidene fluoride (PVDF) membrane and rainbow markers were supplied by GE Healthcare, Amersham, UK. Immobilised m^7^GTP-Sepharose was from Jena Biosciences, Jena, Germany. Human TRAIL was from PeproTech EC Ltd, London, UK. MTT was from Sigma.

### Cell culture

The pancreatic cancer cell lines MIA PaCa-2, BxPC-3 and PANC-1 were all American Type Culture Collection-certified. MIA PaCa-2 and PANC-1 were maintained in Dulbecco’s modified Eagle medium supplemented with penicillin (50 units/ml), streptomycin (50 units/ml) and 10% fetal bovine serum (FBS). BxPC-3 cells were maintained in RPMI 1640 supplemented with antibiotics as above and 20% FBS. Cells were maintained in monolayer cultures at 37 ^o^C in humidified air with 5% CO_2_. MIA PaCa-2 cells with constitutive silencing of 4E-BP1 were engineered using pLKO vectors (Sigma), as previously described^47^. shRNA vector accession numbers are as follows: 4E-BP1 TRCN0000040203 and non-target shRNA control SHC002. Small interfering RNAs targeting 4E-BP1 (Applied Biosystems and Life Technologies, Carlsbad, CA, USA, forward 50-CAAGAACGAACCCUUCCUU-30 and reverse) were transfected using the siPort NeoFx reagent (Applied Biosystems and Life Technologies), according to the manufacturer’s instructions.

### Immunoblotting

Cells were harvested, washed in phosphate-buffered saline (PBS) and subjected to lysis using cell lysis buffer (20 mM Tris-HCl (pH 7.5), 150 mM NaCl, 1 mM EDTA, 1 mM EGTA, 1% Triton, 2.5 mM sodium pyrophosphate, 1 mM β-glycerophosphate, 1 mM sodium orthovanadate (Na_3_VO_4_) and 1 µg/ml leupeptin). Cell pellets were vortexed with buffer and lysed by incubating with lysis buffer on ice for 5 min. Samples were sonicated for approximately five pulses using a sonicator (Jencons), and then centrifuged at 14 000×*g* for 10 min at 4 °C. Equal amounts of whole-cell extract were fractionated by electrophoresis on SDS polyacrylamide gels and the proteins transferred to PVDF paper and immunoblotted as described^63^. Band intensities were determined by quantitative densitometry using ImageJ (http://rsbweb.nih.gov/ij/).

### Tetrazolium reduction assay

Cells were seeded in 96-well plates at 3×10^4^ cells/cm^2^. Following the various cell treatments, 25 μl of MTT were added to each well and left for 2 h in the incubator at 37 °C. The formazan crystals generated by viable cells were solubilized using SDS reagent and cells were incubated overnight in an atmosphere of 5% CO_2_ in a 37 ^°^C humidified incubator. Quantitative determination of cell viability was obtained by utilising a SpectraMax 340PC384 Microplate Reader; absorbance of each sample was measured in quadruplicate at a wavelength of 595 nm.

### Trypan blue exclusion assay

Cells were seeded in triplicate in 12-well plates at 3×10^4^ cells/cm^2^. Following treatment all media and cells were transferred from each well into labelled Eppendorf tubes. A volume of 200 µl per sample were then transferred to fresh Eppendorf tubes with 200 µl 0.4% Trypan Blue solution and tubes were briefly vortexed. Several counts were made for each tube and percentage viability was determined using the following formula: [(number of total cells − number of dead (blue) cells)/number of total (blue and white) cells] × 100 = percentage cell viability].

### Time-lapse microscopy

The kinetics of the commitment of cells to apoptosis were measured by time-lapse digital image microscopy as previously described^64^. Cells were observed in an Olympus IX70 inverted microscope enclosed within a 37 °C chamber in a 5% CO_2_/95% air atmosphere. Images were captured every 15 min using a Hamamatsu C4742-95 digital camera and, for each condition, 40 cells per field of view were randomly chosen at the beginning of the time course. The images were analysed using Image Pro Plus software (Media Cybernetics, USA) with cells committed to apoptosis scored according to the time at which clear changes in morphology (cytoplasmic and nuclear shrinkage and a change to a phase bright appearance) were first observed.

### Flow cytometry

The cells were lifted from the plates with accutase and resuspended in 1 ml cold PBS together with the supernatant media that the cells had been grown in (containing any cells that may have lifted as a result of treatment). Cells were pelleted and the wash repeated. Cells were resuspended in 1× binding buffer at a concentration of 1×10^6^ cells and stained using an FITC Annexin V Apoptosis Detection Kit 1 (BD Pharmingen, San Diego, USA) according to the manufacturer’s instructions. Flow cytometry was carried out on a LSR II flow cytometer (BD Biosciences, San Jose, CA, USA). Analysis was carried out with FlowJo software (Tree Star, Ashland, OR, USA). Unstained cells and cells stained only with FITC Annexin V were used as controls.

### Measurement of overall rates of protein synthesis

Protein synthesis in intact cells was measured by the incorporation of [^35^S] methionine (2–4 μCi/ml for 1 h) into trichloroacetic acid-insoluble material as described previously^54^. Total cellular protein content was determined and overall rates of protein synthesis were calculated as counts per min incorporated per μg protein.

### m^7^GTP-Sepharose chromatography

Initiation factor eIF4E and its associated proteins were isolated from cell extracts (containing equal amounts of protein) by affinity chromatography on m^7^GTP-Sepharose beads as described^65^. Bound proteins were eluted with SDS gel sample buffer and analysed by gel electrophoresis and immunoblotting as described above.

### Statistical analysis

All data are presented as the means ± SEM of at least three independent measurements. Prism 5 software (GraphPad) was used for statistical analysis. A ‘*P*’-value of <0.05 was considered to be statistically significant.

## Electronic supplementary material

Supplementary Figure Legends

Supplementary Figure 1

Supplementary Figure 2

Supplementary Figure 3
