# Supplementary material for: Implication of 4E-BP1 protein dephosphorylation and accumulation in pancreatic cancer cell death induced by combined gemcitabine and TRAIL
Source: Cell Death Dis. 2017 Dec 12;8(12):3204. doi: 10.1038/s41419-017-0001-z (PMC5870593; doi:10.1038/s41419-017-0001-z)
Supplement: Supplementary file 1 — Supplementary Figure Legends [file 41419_2017_1_MOESM1_ESM.docx]

**Supplementary Figures**

**Figure S1 (a)** Effect of gemcitabine and/or TRAIL on PANC-1 cell survival. PANC-1 cells were seeded in 96-well plates at a cell seeding density of 3x10^4^ cells/cm^2^. (**a**) Sensitivity of cells to gemcitabine and TRAIL combination treatment was assessed by MTT assay. Cells were treated with gemcitabine (100 μM) for 24 h (n=4) and 100 ng/ml TRAIL for 2, 4, and 6 h. All experiments were repeated three times and data are provided as means ± SEM. One representative experiment is shown. P-values were calculated using Student’s t test to determine the statistical significance of the difference between cells treated with 100 μM gemcitabine and cells treated with 100 μM gemcitabine and 100 ng/ml TRAIL for 6h (* P <0.05). (**b**) Lysates made from PANC-1 cells treated with or without 100μM gemcitabine for 24 h and/or 100ng/ml TRAIL for 6 h were used to purify eIF4E using chromatography on m^7^GTP-Sepharose beads as described in Materials and Methods. The levels of eIF4E and of the 4E-BP1 associated with it were determined by SDS gel electrophoresis and immunoblotting. Total cell lysates were analysed in parallel. Quantification was carried out by densitometry using Image J and the ratios of 4E-BP1 to eIF4E (in arbitrary units) are indicated for each sample. (**c**) MIA PaCa-2 cells were treated with or without 0.1-100 μM gemcitabine for 24 h and/or 10 or 100ng/ml TRAIL for 4 h. Protein synthesis was determined by labelling with [^35^S] methionine for the last 1 h as described in Materials and Methods. Incorporation of radioactivity is shown as the % of the value for the untreated control cells. The data are the means ± SEM from three independent treatments (** P< 0.01). (**d**) Table showing the % inhibition of protein synthesis following TRAIL treatment alone or in combination with 100 μM gemcitabine. Calculations are based on mean +/- SEM values taken form Figure S1 c.

**Figure S2** Combination treatment targets the mTOR pathway and alters the phosphorylation of 4E-BP1 in PDAC cells. BxPC-3 and MIA PaCa-2 cells were treated with 100μM gemcitabine for 24 h and/or 100 ng/ml TRAIL for 4 h. Fifteen μg of total protein lysate was analyzed using western blotting. (**a**) MIA PaCa-2 cell lysates were analyzed with antibodies directed against total mTOR, mTOR Ser^2448^, Raptor, Rictor, total 4E-BP1, 4E-BP1 Ser^65^ and GAPDH. (**b**) BxPC-3, lysates were analyzed with antibodies directed against total mTOR, mTOR Ser^2448^, Raptor, Rictor, total 4E-BP1, 4E-BP1 Ser^65^ and GAPDH.

**Figure S3** 4E-BP1 is involved in the regulation of cell survival following gemcitabine and TRAIL treatment. (**a**) MIA PaCa-2 cells expressing a small hairpin RNAs (shRNA) directed against 4E-BP1 and control cells expressing a scrambled shRNA were seeded in 96-well plates at a cell seeding density of 3x10^4^ cells/cm^2^. (**a**) The sensitivity of cells to TRAIL treatment was assessed by MTT assay. Cells were treated with increasing amounts of TRAIL (1-1000 ng/ml) for 6 h (n=3). (**b**) The sensitivity of cells to gemcitabine and TRAIL combination treatment was assessed by MTT assay. Cells were treated with increasing amounts of gemcitabine (0.1-100 μM) for 24 h (n=4) and/or 100 ng/ml TRAIL for 6 h (n=4). All experiments were repeated three times and data are provided as means ± SEM. One representative experiment is shown. P values were calculated using Student’s t test to determine the statistical significance of the difference between cells expressing a scrambled shRNA and cells expressing a shRNA directed against 4E-BP1, both cell lines having been treated with 100 μM gemcitabine and 100 ng/ml TRAIL (ns: P>0.05).
