# Supplementary figures and images for: Implication of 4E-BP1 protein dephosphorylation and accumulation in pancreatic cancer cell death induced by combined gemcitabine and TRAIL
Source: Cell Death Dis. 2017 Dec 12;8(12):3204. doi: 10.1038/s41419-017-0001-z (PMC5870593; doi:10.1038/s41419-017-0001-z)

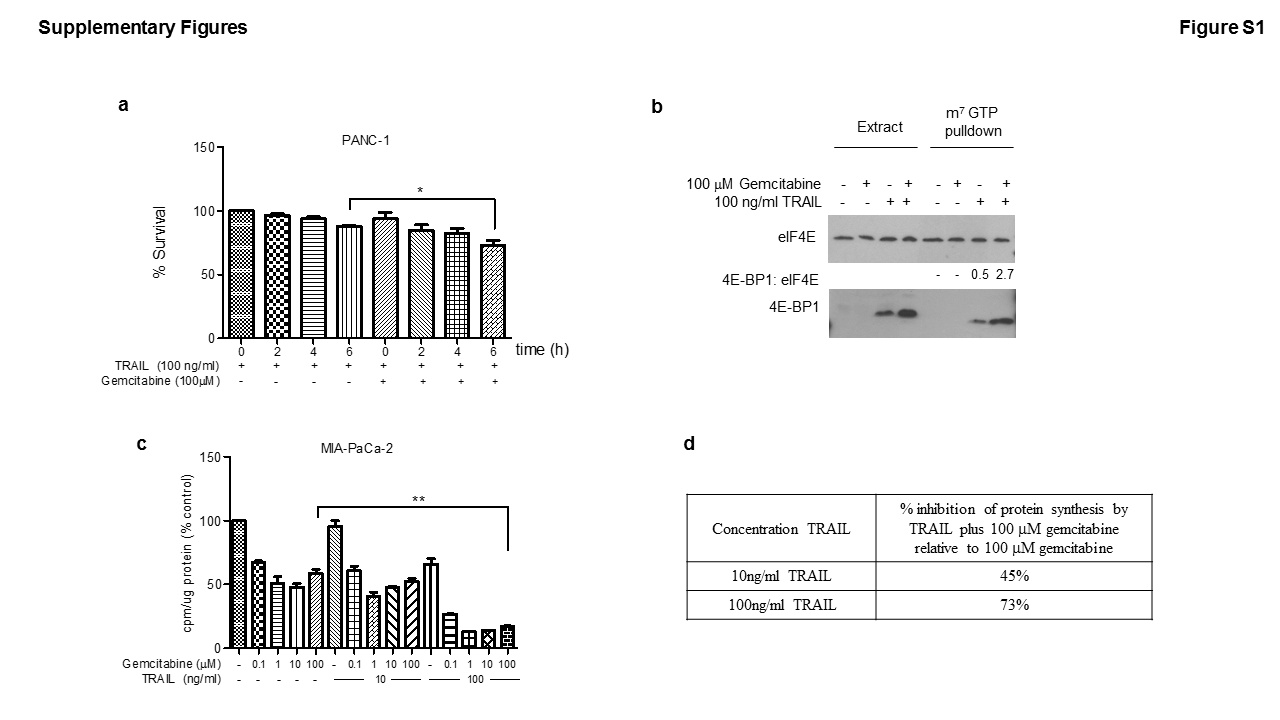

Supplement: Supplementary file 2 — Supplementary Figure 1 [file 41419_2017_1_MOESM2_ESM.tif]

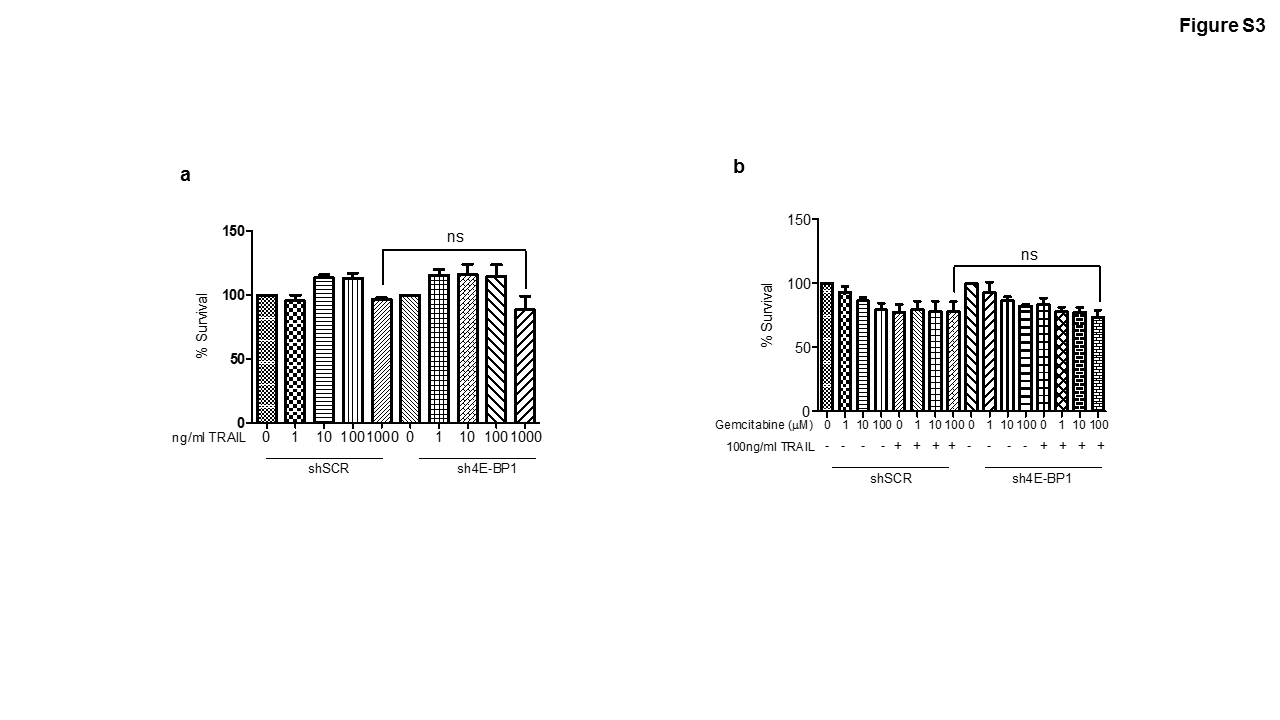

Supplement: Supplementary file 4 — Supplementary Figure 3 [file 41419_2017_1_MOESM4_ESM.tif]
