# Supplementary material for: Implication of 4E-BP1 protein dephosphorylation and accumulation in pancreatic cancer cell death induced by combined gemcitabine and TRAIL
Source: Cell Death Dis. 2017 Dec 12;8(12):3204. doi: 10.1038/s41419-017-0001-z (PMC5870593; doi:10.1038/s41419-017-0001-z)
Supplement: Supplementary file 3 — Supplementary Figure 2 [file 41419_2017_1_MOESM3_ESM.pptx]

## Slide 1
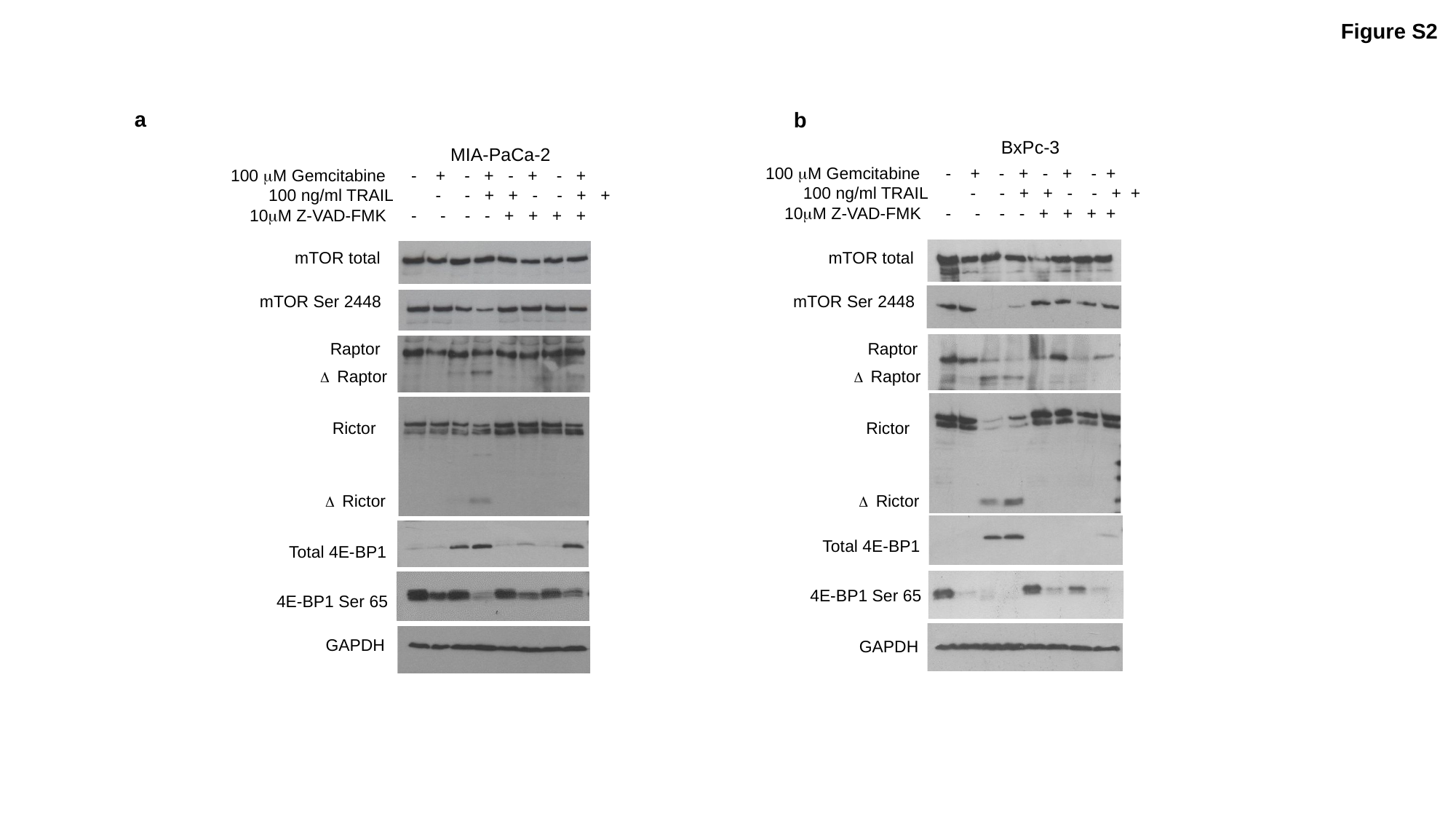

Figure S2
a
b
BxPc-3
 100 mM Gemcitabine	 - + - + - + - +
 100 ng/ml TRAIL - - + + - - + +
 10mM Z-VAD-FMK	 - - - - + + + +
mTOR total
mTOR Ser 2448
Raptor
D Raptor
Rictor
D Rictor
Total 4E-BP1
4E-BP1 Ser 65
GAPDH
MIA-PaCa-2
 100 mM Gemcitabine	 - + - + - + - +
 100 ng/ml TRAIL - - + + - - + +
 10mM Z-VAD-FMK	 - - - - + + + +
mTOR total
mTOR Ser 2448
Raptor
D Raptor
Rictor
D Rictor
Total 4E-BP1
4E-BP1 Ser 65
GAPDH
